# Exercise-Induced Regulation of Redox Status in Cardiovascular Diseases: The Role of Exercise Training and Detraining

**DOI:** 10.3390/antiox9010013

**Published:** 2019-12-23

**Authors:** Tryfonas Tofas, Dimitrios Draganidis, Chariklia K. Deli, Kalliopi Georgakouli, Ioannis G. Fatouros, Athanasios Z. Jamurtas

**Affiliations:** Department of Physical Education and Sports Science, School of Physical Education, Sports Science and Dietetics, University of Thessaly, Karyes, 42100 Trikala, Greece; tryfonastofas@gmail.com (T.T.); dimidraganidis@gmail.com (D.D.); delixar@pe.uth.gr (C.K.D.); kgeorgakouli@gmail.com (K.G.); ifatouros@uth.gr (I.G.F.)

**Keywords:** oxidative stress, redox status, resistance exercise, combined exercise, inactivity

## Abstract

Although low levels of reactive oxygen species (ROS) are beneficial for the organism ensuring normal cell and vascular function, the overproduction of ROS and increased oxidative stress levels play a significant role in the onset and progression of cardiovascular diseases (CVDs). This paper aims at providing a thorough review of the available literature investigating the effects of acute and chronic exercise training and detraining on redox regulation, in the context of CVDs. An acute bout of either cardiovascular or resistance exercise training induces a transient oxidative stress and inflammatory response accompanied by reduced antioxidant capacity and enhanced oxidative damage. There is evidence showing that these responses to exercise are proportional to exercise intensity and inversely related to an individual’s physical conditioning status. However, when chronically performed, both types of exercise amplify the antioxidant defense mechanism, reduce oxidative stress and preserve redox status. On the other hand, detraining results in maladaptations within a time-frame that depends on the exercise training intensity and mode, as high-intensity training is superior to low-intensity and resistance training is superior to cardiovascular training in preserving exercise-induced adaptations during detraining periods. Collectively, these findings suggest that exercise training, either cardiovascular or resistance or even a combination of them, is a promising, safe and efficient tool in the prevention and treatment of CVDs.

## 1. Introduction

Oxidative stress is considered a significant etiological factor for several degenerative diseases, such as cardiovascular diseases [1,2,3,4,5,6], atherosclerosis [7,8], type 2 diabetes mellitus [9,10], cancer [11] as well as neurological diseases with advancing aging [12,13].

**Figure 1:**
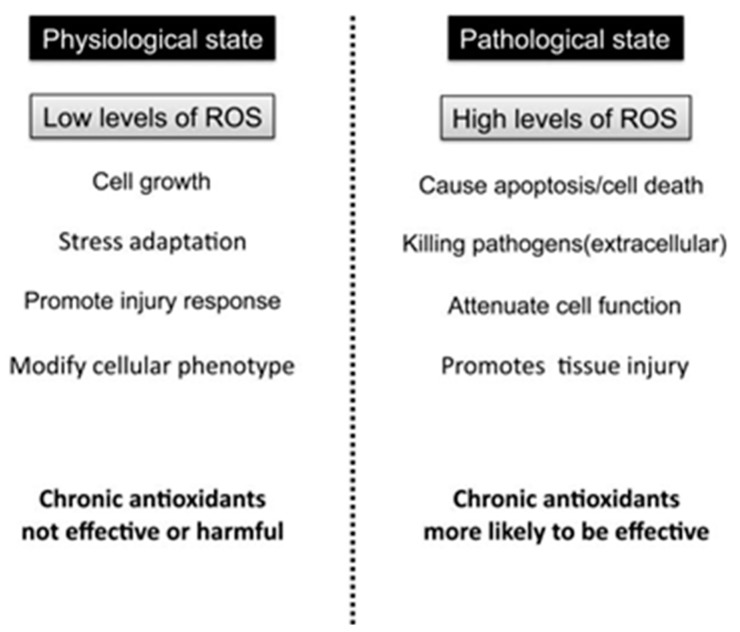
Roles of ROS in physiological vs. pathological state.

It occurs when the production of reactive oxygen species (ROS) exceeds the endogenous antioxidant mechanisms’ ability to counteract them, resulting in redox balance disturbances and oxidative damage to macromolecules [14,15]. Under physiological conditions, low levels of ROS are maintained via the antioxidant defense mechanism eliciting a beneficial effect for the organism, since low ROS concentration is necessary for normal cell and vascular function [16]. Specifically, low levels of ROS can function as specific second messengers for cellular signal transduction pathways, while the balance between oxidizing and reducing species is known to be a crucial regulator of cellular homeostasis [3] (Figure 1). On the other hand, high levels or excess production of ROS, oxidize various molecules, causing damage to lipids, proteins and DNA [16].

Furthermore, numerous reports are indicate that ROS act as a stimulator of inflammation [17,18,19,20]. Actually, the increased production of ROS results in redox-status disturbances promoting the development of a chronic inflammatory response via activation of the redox-sensitive transcriptional factor, Nuclear factor-kappa B (NF-κB), that drives the expression of various pro-inflammatory mediators such as C-reactive protein (CRP) and the cytokines Interleukin-6 (IL-6), Interleukin-1β (IL-1β) and tumor necrosis factor-α (TNF-α) [18,19,20]. Given that chronic inflammation has been associated with the development of numerous diseases such as cardiovascular diseases (CVDs), atherosclerosis, diabetes mellitus (mainly type 2), metabolic syndrome, chronic obstructive pulmonary diseases (COPD) as well as cancer [21,22,23,24], it is evident that oxidative stress plays an important role in the pathogenesis and development of these pathological conditions. Moreover, in terms of CVDs, there is abundant evidence suggesting that oxidative stress is the driving force for atherosclerosis, ischemia-reperfusion injury, chronic ischemic heart disease, cardiomyopathy, heart failure, hypertension, myocardial infraction, angina pectoris, and even ensuing arrhythmias [25,26].

To date, although it has been established that oxidative stress plays major role in the development of CVDs, large interventional studies have failed to consistently show beneficial effects after antioxidant supplementation in preventing or treating CVDs [27,28,29]. One possible reason might be that ROS are not universally harmful since repeated, low-level exposure to ROS is a vital trigger for up-regulating endogenous antioxidants [30]. Thus, researchers have directed their attention towards non-pharmacological therapies in order to reduce oxidative stress focusing mostly on exercise training and physical activity. In the present review, we provide an overview of the role of oxidative stress in the onset and progression of CVDs and outline the available evidence regarding the effects of acute and chronic exercise training and physical activity interventions on oxidant/antioxidant status in the context of CVDs. The role of detraining and physical inactivity is also discussed.

## 2. Oxidative Stress and Cardiovascular Disease

CVD and endothelial dysfunction are characterized by a chronic inflammatory response and oxidative stress [31,32]. Oxidative stress is the driving force in the pathogenesis and development of most CVDs, predisposing individuals to atherosclerotic manifestations and cardiovascular complications such as atherosclerosis, hypertension, ischemic heart disease and cardiac myopathy [33].

Elevated ROS levels has been shown to contribute to vascular dysfunction both in animal models and clinical studies [34,35,36,37]. According to Ungvari et al. [35], ROS-mediated activation of retrograde signaling pathways, including NF-κB, lead to chronic low-grade systemic inflammation promoting the development of vascular diseases in the elderly. Furthermore, Machi et al. [38] suggested that impairment of heart function may be related to increased oxidative stress in the tissue. While low levels of ROS are considered beneficial for various biological processes such as endothelial function, vascular tone and cardiac function, when excessively produced, ROS, can disrupt cellular signaling and induce cellular damage [25]. Indeed, enhanced activation of oxidative stress and inflammatory mediators can either directly cause injury to myocardium or increase the atherosclerotic process [39]. Specifically, in CVDs where generation of ROS is increased and the renin angiotensin system is often upregulated, these redox-sensitive events may contribute to cellular processes involved in vascular dysfunction and structural remodeling [40].

Lipid peroxidation and protein oxidation due to increased ROS production, result in overexpression of redox genes, intracellular calcium overload and DNA fragmentation, causing damage to vascular smooth muscle cells (VSMCs), endothelial cells or myocardial cells [37,41,42,43]. In addition, lipid peroxidation is also actively involved in the peroxidative modification of low-density lipoprotein (LDL) [37,41,42,43], that when oxidized (i.e., oxidized LDL) plays a key role in the development of atherosclerosis [44]. Moreover, Zembron-Lancy et al. [32], have recently shown that levels of oxidized low density lipoprotein (oxLDL), protein carbonyls (PC) and lipid peroxidases (LPO) were elevated in elderly men and were highly correlated with common CVD factors such as LDL, high-density lipoprotein (HDL) and Framingham score. Thus, a vicious cycle of oxidative stress and oxidative stress-induced atherosclerosis is evident leading to development and propagation of atherosclerosis [45].

Detection and measurement of circulating biomarkers of oxidative stress though, is a challenging process, since blood circulation may function independently of the vascular wall’s individual structures [15]. Nevertheless, it has been noticed that several markers of oxidative stress in plasma were increased in CVD patients [46], while plasma levels of oxLDL is considered to be a prognostic indicator of mortality in subjects with congestive heart failure [46,47]. In addition, many oxidative stress biomarkers, such as serum lipid hydroperoxides, plasma malondialdehyde (MDA) or urine F2-isoprostanes, are utilized as a prognostic tool for assessing the risk of CVDs [15,41]. A recent study showed a significant increase in MDA, oxLDL and PC, and a significant decrease in superoxide dismutase (SOD) activity, in menopausal women with CVD as compared to their healthy counterparts [41].

Thus far, it is well-documented that multiple factors are involved in the pathophysiology of CVDs and oxidative stress is undoubtedly a strong contributor to the atherogenic process [30]. Treatments with dietary supplements and antioxidants aiming at reducing or alleviating the damaging effects of oxidative stress have long been considered as potential strategies to prevent or reduce CVD progression. With this aim antioxidant substances have received far more attention, due to their direct influence on antioxidant capacity and oxidative stress levels [30].

## 3. Antioxidants, Oxidative Stress and Cardiovascular Disease

Antioxidants are substances that offer protection against a wide spectrum of diseases, due to their ability to neutralize free radicals produced by either endogenous metabolic processes or exogenous sources, preventing as such the ROS-mediated damage to lipids, proteins and DNA and providing cellular protection [30]. The enzymatic and non-enzymatic antioxidant systems are the primary antioxidant mechanisms in human body that work synergistically to protect cells and organ systems against ROS-mediated damage [6,48,49,50]. The former includes endogenously produced substances such as the enzymes catalase, glutathione peroxidase (GPx) and SOD, while the latter comprises substances derived from diet and dietary supplements such as vitamins (A, C and E), polyphenols, carotenoids as well as the *N*-acetylcysteine [51,52,53].

Interestingly though, the available evidence from interventional trials [6,30] have failed to confirm the beneficial effects of antioxidant supplements administration on CVDs, reported by previous observational cohort studies [54]. Specifically, according to a literature review performed by Chen and colleagues [6], oral antioxidant supplements have been ineffective as either preventative or therapeutic agents in CVDs. Furthermore, associations between plasma concentrations of antioxidant vitamins (A, C and E) and protection against cardiovascular disease have shown to be elusive and large interventional studies that used these vitamins have failed to conclusively show any benefit [30].

In contrast to antioxidant supplements, chronic exercise training has been characterized as a non-pharmacological tool to prevent or treat many CVDs since it elicits cardio-protective effects via multiple mechanisms. Specifically, there has been reported that regular exercise is capable of improving insulin sensitivity and lipid metabolism, regulating autonomic function [55], reducing blood pressure and blood viscosity, enhancing endothelial nitric oxide production as well as improving leptin sensitivity [56]. Moreover, exercise increases the mitochondrial biogenesis, fatty acid oxidation and the dilation of blood vessels, that results in improved myocardial perfusion and lower inflammation, reducing as such the risk for atherosclerosis [57]. In particular, regular exercise offers indirect antioxidant protection by enhancing the activity of the endogenously produced antioxidant enzymes [58,59,60]. It should be highlighted though, that exercise induces intensity-, duration- and type-dependent effects on antioxidant mechanisms and oxidative stress levels. Very intense or exhaustive exercise (either acute or prolonged) increases oxidation and downregulates the antioxidant defense resulting in excessive damage to macromolecules [61,62], whereas low-to-moderate intensity exercise (particularly repeated exercise bouts) enhances the activity of antioxidant enzymes and consequently lowers the levels of generated ROS and improves the cellular adaptation to subsequent stress [63,64]. For instance, a significant decrease in SOD, GPx, and catalase activity has been reported following exhaustive high intensity exercise [61,62], while an 8-week training program of moderate intensity resulted in improved total antioxidant capacity and reduce lipid oxidation [65].

Therefore, in an attempt to enhance our understanding of how exercise training regulates oxidative stress in CVDs, we provide a thorough review of the related literature in the following sections by presenting all the available evidence regarding the effect of exercise training mode/type (cardiovascular vs. resistance vs. combined), intensity (low vs. moderate vs. high) and duration (acute vs. chronic/regular vs. inactivity).

## 4. Exercise, Cardiovascular Disease and Oxidative Stress

Numerous epidemiological studies have convincingly demonstrated the beneficial effect of physical exercise on CVD outcomes, and thus, it has been considered as a valuable therapeutic approach for CVDs [56,66,67,68,69]. The favorable effect of regular exercise on CVDs progress is primarily attributed on the exercise-mediated enhancement of the antioxidant capacity and reduction of oxidative stress levels that subsequently results in redox balance preservation and cellular homeostasis [63,64].

Indeed, there has been documented that regular exercise increases the expression of major antioxidant enzymes and reduces that of pro-oxidant ones [70]. A recent study has shown that 8 weeks of cardiovascular exercise (CVE) training, enhanced the total antioxidant capacity (TAC) and reduced MDA levels in myocardium while it also ameliorated the cardiac damage induced by oxidative stress in ovariectomized rats [65]. The improved oxidative stress status induced by exercise was associated with cystathionine-γ-lyase expression (CSE) in myocardium, suggesting that this improvement might be at least partially due to the upregulation of CSE expression [65].

As reviewed by Radak and his colleagues [64], regular exercise plays a preventive role against ROS-dependent diseases and the molecular mechanism underlying this favorable effect could be linked to redox status homeostasis. In fact, the exercise-induced activation of transcriptional factors promotes a molecular cascade that results in increased activation of antioxidant enzymes, DNA repair enzymes and the ubiquitin-proteasome system [64]. Subsequently, these cellular events lead to an improved physiological function and enhanced resistance to oxidative stress [64].

In addition, another proposed mechanism through which exercise regulates lipid metabolism and promotes antiatherogenic benefits, is the ligand-depended activation of the redox-sensitive transcription factors, peroxisome proliferator activated receptor γ (PPARγ) and liver X receptors (LXRs) [71,72,73]. Specifically, the exercise-mediated generation of free radicals increases the circulating levels of oxidized low-density lipoprotein (oxLDL) resulting in activation of PPARγ and LXRα in monocytes, and subsequently increased expression of their target genes, CD36, ABCA1 and ABCG1 [71,72,73]. The transcriptional activation of CD36 by PPARγ promotes the cellular uptake of oxLDL while the upregulation of ABCA1 and ABCG1 by LXRα enhance the reverse cholesterol transport, thus inducing beneficial effects to blood lipid profile by increasing the HDL cholesterol levels and reducing that of LDL, total cholesterol and triglycerides [72]. The paradoxical here is that, although it is well-described that free radicals are associated with increased oxidative stress and damage to macromolecules following an acute bout of intense or exhaustive exercise [74] and they are also critically implicated in the pathogenesis of CVDs [33] and age-related chronic diseases [12,13], the aforementioned mechanism suggests that generation of low levels of free radicals in response to regular exercise is essential for an antiatherogenic effect to occur. The rationale for this phenomenon is the ″hormesis‶ theory for the exercise-mediated production of ROS, proposed by Radak et al. [75]. According to this theory, participation in regular exercise promotes transient increases in ROS formation that evoke adaptive responses rather than damaging effects, and activate cellular signal transduction pathways that promote beneficial adaptations. The ″hormesis‶ theory may also explain the blunted activation of PPARγ and LXRα observed when exercise is accompanied by dietary antioxidant supplementation [76,77,78]. It is the antioxidant-mediated reduction in exercise-induced generation of free radicals that leads to lower oxLDL generation and subsequently to downregulation of PPARγ and LXRα, diminishing as such the associated health-related benefits [76,77,78].

### 4.1. Effect of Exercise on Hypertension and Oxidative Stress

The pathogenesis of hypertension is a complex process, involving, amongst others, regulation of kidney NaCl handling, alterations to arterioles and regulator mechanisms in the brain [79]. Both exercise and physical activity promote a blood pressure lowering effect through various mechanisms [79,80]. For instance, the exercise-mediated reduction in peripheral vascular resistance, sympathetic nervous system activity and renin-angiotensin-aldosterone that result in prevention of left ventricular hypertrophy, is a proposed mechanism [79]. In addition, the favorable changes occurred in inflammatory status, endothelial function, arterial compliance, body mass, renin-angiotensin system activity, parasympathetic activity, renal function, insulin sensitivity and oxidative stress, in response to exercise, are also considered potent mechanistic links between exercise and lower blood pressure [80]. However, in the present review we focus on the available evidence examining the effect of exercise on hypertension through oxidative stress regulation.

Oxidative stress has been repeatedly associated with human essential hypertension [26,81]. Notably, numerous clinical studies provided evidence that ROS production is elevated in hypertensive patients, since they have detected increased plasma levels of oxidative stress biomarkers related to protein and lipid oxidation such as thiobarbituric acid-reactive substances (TBARS) and 8-epi-isoprostanes, while at the same time the activity of antioxidant enzymes such as SOD, GPx and catalase was substantially decreased [82,83,84]. Therefore, a notable reduction in ROS production mediated by the enhanced level of antioxidant protection has been proposed to be a major mechanism through which exercise lowers blood pressure [85].

Exercise training has been widely recommended as an effective non-pharmacological therapeutic approach for hypertensive patients [86,87,88]. The beneficial effect of exercise on these patients is primarily attributed on the decreased levels of oxidative stress and the improved redox status [87,89,90] that have as a consequence the reduction of blood pressure [85].

Specifically, Trape and colleagues [89] reported that a higher level of training status is associated with improved nitrite concentration as well as systolic and diastolic blood pressure, suggesting that the mechanism underlying the control of blood pressure includes a higher antioxidant capacity achieved by higher level of training status and consequently, higher nitric oxide bioavailability. Furthermore, exercise training at a moderate intensity has a beneficial effect in preventing the development of hypertension by lowering inflammatory cytokines and thus preventing pathological changes to vessel cells and normalizing changes in blood pressure [87]. Also, Cook and colleagues [91] have shown that resistance exercise (RE) training is an effective type of exercise in modulating matrix remodeling proteins and oxidative stress, strengthening as such the role of RE training in the potential prevention of the early onset of hypertension. These findings are consistent with recent evidence that CVE training of moderate intensity and interval training reduces blood pressure and also provides a favorable change in antioxidant status by reducing levels of MDA in plasma [92,93].

Moreover, exercise training has been reported to increase nitric oxide (NO) production and decrease NO inactivation, leading to increased NO bioavailability and improved endothelial function in animal models of hypertension and in patients with essential hypertension [86]. These findings suggest that endothelial dysfunction in hypertension is reversible, and that regular exercise may safely and efficiently utilized to combat the elevated blood pressure. A previous study in rats has shown that 12-weeks of low-intensity CVE training decreases oxidative stress and increases NO bioavailability, allowing a complete reversal of the augment contractile response observed in small mesenteric arteries [94]. Thus, it has been suggested that the exercise-induced reduction of oxidative stress is responsible for the improvement in coronary artery endothelial dysfunction in hypertension [94].

In addition, ten weeks of swimming training, reduced cardiac oxidative stress, exacerbated cardiac hypertrophy, improved ventricular function, induced resting bradycardia and decreased blood pressure in spontaneously hypertensive rats [95]. Although it seems paradoxical that swimming training enhanced cardiac hypertrophy in this study, a previous observation that endurance training promotes cardiac performance by converting the pathological cardiac hypertrophy, induced by hypertension, into physiological hypertrophy [96], provides an explanation of this finding. A previous study also reported that a 3-week intervention with low-fat, high-fiber diet combined with low-intensity CVE training induced a remarkable improvement in blood pressure, oxidative stress and NO availability, in hypertensive patients [97]. Likewise, Agarwal et al. [98] reported that 4 weeks of moderate-intensity CVE training in hypertensive rats not only reduced blood pressure and improved cardiac function but also reduced inflammatory cytokines and norepinephrine, diminished activation of NF-κB and decreased oxidative stress, as indicated by the reduction in inducible nitric oxide synthase (iNOS) expression and the increased levels of cooper/zinc containing superoxide dismutase (Cu/ZnSOD) within the paraventricular nucleus (PVN). PVN is a crucial brain region that serves as an autonomic control center regulating cardiovascular, neuroendocrine and physiological functions [99,100]. Alterations in PVN neurons can influence the sympathetic outflow in hypertension by modulating vasopressin release while exercise training has been proposed to affect PVN neuronal activity by preserving their normal function in hypertension [99,100].

Although Sturgeon and colleagues [101] noted that changes in cholesterol levels but not oxidative stress or endothelial biomarkers were related to changes in blood pressure following 6 months of moderate CVE training, a previous study indicated that improvement of the enzymatic antioxidant defense resulted in reduced mean arterial blood pressure (MABP) [102]. In addition, the authors reported that MABP was correlated with both nitrotyrosine and 8-hydroxyl-2′-deoxyguanosine (8-OHdG), with a 25% reduction of oxidative stress inducing a decrease in MABP by 10 mm Hg [102].

Collectively, these data indicate that exercise training decreases blood pressure and improves endothelium-dependent vasodilation in hypertensive patients through the increased bioavailability of NO in the vascular wall.

### 4.2. Effect of Exercise on Heart failure and Oxidative Stress

Oxidative stress has been shown to play a crucial role in the pathophysiology of cardiac remodeling and development of heart failure (HF) [70,103,104,105], causing cardiomyocyte death, abnormalities in transduction of myocardial β-adrenergic receptor signaling and contractile dysfunction [106,107]. As reviewed in the recent work by Heinonen and colleagues [108], the increased extravascular compressive forces and coronary microvascular dysfunction developed in the failing heart induce an imbalance to myocardial oxygen.

Chronic HF is also associated with increased oxidative stress [109], as indicated by reduced levels of antioxidants, redox status disturbances and increased lipid peroxidation in HF patients [110]. Previous studies have shown that, oxidative stress markers such as lipid peroxides and levels of 8-OHdG are elevated in serum and urine of patients with HF, with the urinary 8-OHdG levels reflecting the clinical severity of HF on the basis of symptomatic status and cardiac dysfunction [110,111,112,113]. Furthermore, the activity of antioxidant enzymes such as serum paraoxonase-1 (PON-1), myocardium manganese superoxide dismutase (MnSOD), SOD, calatase, GPx and thioredoxin reductase are diminished in HF patients [113,114]. In addition to redox status perturbations and oxidative stress, the presence of increased levels of pro-inflammatory cytokines as well as low levels of anti-inflammatory cytokines is also evident in patients with HF [105,115], suggesting therefore, that HF is characterized by a pro-oxidant and pro-inflammatory response that regulates the onset and progression of the disease.

Extensive research on this field has demonstrated that exercise training offers multiple benefits in HF patients, both at clinical and molecular level, and thus, engagement in regular exercise has become a class I recommendation in all national and international guidelines for the prevention and treatment of chronic HF [116]. Indeed, participation in regular exercise has been proposed to enhance the patients’ physical performance and their quality of live, by improving parameters related to skeletal muscle, cardiovascular system and endothelial function as well as by promoting metabolic and neurohumoral adaptations [117]. One of the most striking exercise-mediated benefits in HF is the decrease in sympathetic nervous activity [118,119], associated with decreased oxidative stress, pro-inflammatory cytokines [120] and iNOS expression [121,122]. Negrao and colleagues [119] reported that only exercise training has been shown to be able to restore the neurohormonal balance and reverse many key features of the skeletal myopathy in patients with HF from systolic dysfunction. In addition, both animal-based research and human studies, have demonstrated that physical exercise is a powerful signal that improves peripheral function in chronic HF and increases the mitochondrial volume and enzyme content, improving the metabolic capacity [119,123,124]. Moreover, regular exercise enhances the endothelial function via an increase in shear stress that promotes nitric oxide synthase (NOS) expression and activity, resulting in more bioavailable NO [123]. Koba et al. [124] suggested that oxidative stress in the medulla mediates central command dysfunction, while exercise training in chronic HF is capable of normalizing central command dysfunction through its antioxidant effects in the medulla.

An antioxidant effect is also induced by exercise training in HF via reduction of NAD (P)H oxidase (gp91^phox^, p22^phox^, and Nox4) and augmentation of the activity of radical scavenger enzymes [70,125,126]. Specifically, Gao et al. [70] showed that CVE training normalized the sympathetic outflow and arterial baroflex function in chronic HF rabbits, that was mediated via regulation of SOD expression and downregulation of gp91^phox^ expression in rostral ventrolateral medulla. More recently, in the work by Zucker et al. [123] it was reviewed that exercise training reduces sympathetic outflow in HF state and components of the renin ANG II system, with ROS playing an important role in this process both in the central nervous system and the periphery nitric oxide (NO).

Eight weeks of CVE training (swimming) enhanced the left ventricular end-diastolic pressure, increased the levels of the anti-inflammatory cytokine IL-10, reduced TBARS in skeletal muscle and decreased lipid peroxidation in Wistar rats with chronic HF [115]. Likewise, CVE training over an 8-week period attenuated cardiac endoplasmic reticulum stress in post-myocardial infraction HF rats by recovering the cardiac proteasome activity, which is related to improved left ventricular (LV) function and exercise capacity [127]. Gomes et al. [109], also showed that 8 weeks of low-intensity exercise training improved cardiac structure and function, reduced oxidative stress, preserved antioxidant enzyme activity, reduced the expression of total c-Jun NH2-terminal kinase (JNK) and increased the phosphorylation of extracellular signal-regulated kinase (ERK) 1/2 in rats with aortic stenosis-induced HF, without any changes observed either in NADPH oxidase activity or NF-κB pathway protein expression. Furthermore, mice with clinical sings of HF exhibited reduced lipid hydro peroxides, restored iNOS expression and increased activity of citrate synthase in red portion of gastrocnemius paralleled by increased capillaries per muscle fiber, following 8 weeks of running at a maximal lactate steady-state workload [121]. In line with these findings, improved cardiac function, resistance to oxidative stress-induced cell death, increased systolic calcium ions (Ca^2+^) transient amplitude and improved diastolic Ca^2+^ removal have been also reported in HF rats following CVE interval training lasted for eight weeks [128].

In a human study, eight weeks of unsupervised, home-based exercise training induced a significant improvement in physical work capacity and led to a normalization of hypoxanthine levels (a pro-oxidant substrate and a marker of hypoxia) in patients with stable HF [129]. In addition, Tsarouhas et al. [130] reported that unsupervised, daily physical activity of moderate intensity was able to ameliorate the lipid and glycemic profile of HF patients, with simultaneous attenuation of inflammation and oxidative stress. According to these findings, 6 months of regular CVE training increased catalase and GPx activity in skeletal muscle by 42% and 41% respectively, and decreased lipid peroxidation by 57% in HF patients [125]. However, the authors noted that despite the augmentation in catalase and GPx levels, exercise training failed to affect total SOD activity, suggesting a persistent impairment of superoxide radical detoxification in the skeletal muscle [125]. Hence, these results suggest that exercise training represents a costless and effective therapeutic strategy for HF, by preserving redox status and cellular homeostasis.

The available evidence regarding the effects of exercise on HF patients with diastolic and systolic HF is limited and equivocal. Klempfner et al. [131] reported that a 6-month structured exercise training program induced comparable improvement in exercise capacity in both HF patients with preserved ejection fraction (exhibit abnormal diastolic function) and those with reduced ejection fraction (exhibit abnormal systolic function). On the other hand, there has been shown that despite the increase in cardiac output and preservation of low-left ventricular diastolic pressure were equally diminished in HF patients with preserved and reduced ejection fraction during a graded exercise protocol on a treadmill, there was a substantial difference among the two groups in the response of left ventricular systolic and diastolic pressure to exercise [132]. The authors concluded, that the pathophysiological mechanisms underlying HF with preserved and HF with reduced ejection fraction differ substantially, and therefore the exercise-mediated effects on these clinical conditions are also distinguishing [132].

To conclude, it should be highlighted that most of the current evidence regarding the effect of exercise training on HF patients relies on data from patients assigned as either functional class II or III, according to the New York Heart Association (NYHA) [133,134]. There is limited information regarding the beneficial role of exercise in patients with unstable HF or those categorized as NYHA functional class IV [133,134]. Therefore, it reasonable that exercise prescription is currently proposed for NYHA class II and III HF patients and to NYHA class IV ones who are asymptomatic in the resting state [133].

### 4.3. Effect of Exercise on Atherosclerosis and Oxidative Stress

Atherosclerosis is a chronic inflammatory disease of arteries, associated with blood lipid disorder, mitochondrial DNA damage and oxidative stress [34]. Accumulating evidence suggests that endothelial dysfunction is an early event in the progression of atherosclerosis [135]. Notably, the dysregulation of the endothelial lining occurred in the lesion-prone areas of the arterial vasculature has been proposed to be a major stimulator of the atherosclerotic process [136].

Enhanced lipid peroxidation and decreased antioxidant protection, predominantly found in metabolic diseases, or unhealthy lifestyle can induce endothelial dysfunction and atherosclerosis [137]. According to Davignon and Ganz [138], a defect in the production or activity of nitric oxide leads to endothelial dysfunction, triggered by impaired endothelium-dependent vasodilation. Nitric oxide counteracts the effect of endothelium-derived vasoconstrictors and inhibits oxidation of low-density lipoprotein [138]. Clinical studies have shown that formation of ROS is a critical event in the development of atherosclerosis [110] and upregulation of oxidative stress and downregulation of the antioxidant defense mechanism are evident in subjects with coronary artery disease (CAD) [137]. Therefore, regulation of redox status and metabolism of lipoproteins have been proposed as two potential mechanisms through which exercise promotes beneficial effects on atherosclerosis [139].

Various types of exercise training are able to induce beneficial effects on the progression of subclinical atherosclerosis [140,141,142,143]. Regular physical activity not only improves clinical symptoms but also affects the progression of atherosclerosis by reducing the rupture of atherosclerotic plaque and enhancing the endothelial-related relaxation, which is protective against endothelial NOS (eNOS-NO) dysfunction in atherosclerosis [135,140,144,145,146,147]. These effects are at least partially attributed to improved endothelial dysfunction mediated by increased NO bioavailability [148].

In addition, a recent study showed that 12 weeks of voluntary wheel running training reduced atherosclerotic plaque area in apolipoprotein E Knockout (apoE^−/−^) mice with 5/6 nephrectomy [144]. Similarly, Kadoglou at al. [145] reported that following 6 weeks of treadmill training, vulnerable atherosclerotic plaques were stabilized via the modulation of inflammatory pathways and matrix metalloproteinases in diabetic apoE^−/−^ mice. Moreover, 4 weeks of treadmill training in low lipoprotein receptor knockout (LDLr^−/−^) mice resulted in reduced atherosclerotic plaque formation that was attributed to modulation of lipid metabolism, possibly by stimulating cholesterol reverse transport lipoprotein genes and through a set of anti-inflammatory cytokine genes [149]. These results are in agreement with a previous study, reporting that swimming training for 8 weeks reduced atherosclerosis by providing increased antioxidant protection via the vascular NO system, in apolipoprotein E deficient mice [146].

The role of combined exercise and diet on lipid profile, inflammation and atherosclerosis [150] as well as their association with oxidative stress [151], has been also examined. Specifically, Lee et al. [151], utilized a combination of swimming training and dietary supplementation over an 8-week period, reporting that oxidative stress was reduced by reducing 4-hydroxynonenal (4-HNE), aorta vasodilatation was enhanced through increased NO and eNOS expression in aorta, while CRP and pro-inflammation proteins were reduced, in aged rats with diet-induced atherosclerosis.

Previous studies proposed that increased activation antioxidant enzymes may reduce atherosclerosis through the co-activation of vascular relaxation mediated by NO, as the superoxide (O_2_^−^) dismutation increases the bioavailability of NO in endothelial cells and in the presence of the O_2_^−^ NO shifts to formation of peroxynitrite (ONOO^−^) [137,152,153]. Furthermore, according to Teodoro et al. [139], moderate- and low-intensity CVE training performed daily for 30 min over an 8-week period, increased the activity of the antioxidant enzymes SOD and GPx, decreased lipid hydroperoxides and PC formation and decreased atherosclerotic lesions in mouse models with atherosclerosis.

## 5. Cardiovascular Exercise and Oxidative Stress

Advanced research has indicated that signaling pathways that are actively involved in the regulation of mitochondrial oxidative stress, biogenesis and function, emerged as potential therapeutic targets for the amelioration of vascular dysfunction and prevention of age-related vascular diseases [35,154]. According to Chen et al. [155], protection of mitochondria from bioenergetics failure and oxidative stress, factors that lead to apoptosis in the ischemic tissue, may open a new vista to the development of more effective neuroprotective strategies against ischemia-induced brain damage. Furthermore, a previous study suggested that low oxidative stress levels promote the expression of intracellular antioxidants resulting in enhanced myocardial tolerance to ischemia [156]. Therefore, these data strongly suggest that myocardial adaptation to oxidative stress may be a potential tool for counteracting the ischemic/reperfusion injury.

Hypothetically, an increased rate of electron flow via the mitochondrial electron transport chain caused by increased oxygen consumption during CVE, could amplify free radical production. In the resting state, ROS are produced at a rate that the antioxidant defense mechanism is able to compete with, preserving their levels at normal ranges [157]. During CVE though, ROS production increases in parallel with oxygen consumption [158] while the antioxidant system is launched to maintain redox balance [159]. However, CVE improves mitochondrial function, increases the number of muscle mitochondria [160,161,162] and results also in enhanced adaptation to oxidative stress by increasing the level of antioxidants [85]. Therefore, it is evident that CVE increases both ROS production and the antioxidant enzyme activity, promoting a net effect of improved efficiency of the antioxidant defense mechanism despite the increased oxygen consumption [159,163]. Given that the effects of regular exercise differ substantially from those induced by acute exercise bouts, we present these exercise modes separately in the next paragraphs.

### 5.1. Acute Cardiovascular Exercise

Repeated bouts of sustained and/or high intensity CVE, such as that required for marathon training and competition, evoke systemic vascular remodeling that shifts the effect of CVE from cardio-protective to atherogenic [164]. Mastaloudis et al. [157] have shown that extreme endurance exercise promotes lipid peroxidation with concomitant increased vitamin E disappearance. In addition, Kliszczewicz et al. [165] proposed that the oxidative stress response is proportional to the exercise intensity performed. In this study, following an acute bout of exercise (at 90% of maximum heart rate (HRmax)), plasma lipid hydroperoxides (LOOH) and ferric-reducing antioxidant power (FRAP) were increased immediately post exercise and remained elevated as far as 2 h post-exercise, whereas PC and trolox-equivalent antioxidant capacity (TEAC) were decreased immediately post exercise and remained low for 1–2 h following exercise, in healthy individuals. Similarly, another study also showed that a single bout of prolonged, moderate-intensity CVE (3-h walk at 30% of heart rate reserve) did not increase the lipid peroxidation levels, whereas an increase in the antioxidant defense was observed at the end of the exercise [166]. Furthermore, there has been reported that a single bout of high-intensity interval training (8x1min at 100% or 90% of Peak Power on a cycloergometer), promotes lymphocyte oxidative stress and reduces lymphocyte proliferation in response to super-antigenic stimulation [167].

The work by Brito et al. [168,169] has indicated that exercise intensity regulates the lipid peroxidation in the heart, aorta, lung and trachea in Wistar rats. Actually, it was observed that low-intensity swimming exercise promotes acute vasorelaxant activity and increases lipid peroxidation, whereas high-intensity swimming exercise (above the anaerobic threshold) reduces the relaxant effect of exercise and leads to a further elevation in lipid peroxidation [168,169]. A previous study though, reported that regular, low-intensity CVE prevents the oxidative stress response to an acute bout of exhaustive CVE, by upregulating the antioxidant enzymes’ activity [170]. In addition, Seifi-skishahr and colleagues [171] did recently suggest that the effect of a high-intensity exercise bout on glutathione redox ratio depends on the individual’s physical conditioning status and also, proposed that engagement in exercise training of moderate intensity may improve health by shifting the “redox” balance towards a more reduced environment, encountering stressful conditions. The authors also provided evidence that training status affects the GSH/GSSG ration in plasma and red blood cells, and the cysteine/cystine (Cys/CySS) ratio in plasma, both in the basal state and following exercise. Table 1 lists all studies that examined the effects of acute CVE on redox status.

### 5.2. Regular Cardiovascular Exercise Training

Maximal oxygen consumption (VO_2_max) has been recently shown to be positively correlated with total antioxidant status [32]. However, the exercise-mediated adatations to antioxidant defense mechanism occurs progressively over time, as both ROS generation and markers of oxidative stress are elevated within the first few weeks of low-intensity exercise [163]. Therefore, it seems that prolonged periods of CVE training are required for the improvement of the antioxidant system and downregulation of the free radical generation.

Animal-based studies have consistently demonstrate that regular CVE training enhances the activity of endogenous antioxidants and reduces the levels of oxidative stress and inflammation in various tissues, particularly in skeletal and cardiac muscle [183,184,185,186,187,188,189]. For example, Chis et al. [183] showed that participation in a 4-week programme of moderate-intensity swimming, resulted in improved hyperglycemia, hypertriglyceridemia, hypercholesterolemia and antioxidant status as indicated by the increased levels of SOD and catalase and decreased MDA, PC, NOx and iNOx levels, in aortic tissue of diabetic Wistar rats. Likewise, 8-weeks of moderate-intensity swimming diminished heart expression of lectin-like oxidized low-density lipoprotein receptor-1 (LOX-1) and reduced oxidative stress levels in rats receiving a high-fat diet [185]. In addition, Coelho et al. [186] provided evidence that CVE training is able to prevent atrophy, oxidative stress and muscle damage in skeletal muscle of rats characterized by sepsis. The authors concluded that the exercise-mediated enhancement of the antioxidant defense mechanism in muscle is most likely the underlying mechanism for the protection of muscle cells against oxidative damage [186]. Notably, a 6-week intervention with self-estimated, moderate-intensity CVE training, resulted in positive changes in MDA, SOD, GPx and catalase activities, in the heart tissue of Wistar rats [187], suggesting that even exercise at a voluntary-adjusted intensity is an efficient strategy for improving heart function.

In line with these findings, the recent work by Gimenes et al. [184] indicated that low-intensity CVE training over a 9-week period, prevents the increase in myocardial lipid peroxidation and attenuates the reduction in antioxidant enzyme activity in a rat model of type 1 diabetes. Moreover, Holland et al. [188] reported that 10 days of moderate-intensity treadmill training in rats, up-regulated the activity of the main endogenous antioxidant enzymes and decreased inflammatory mediators in ileum tissue at 24h post-exercise. A similar response was also noted following 16 weeks of moderate-intensity endurance training characterized by a significant increase in SOD1 and catalase in intestinal lymphocytes [189].

Long-term interventions in humans support the previous findings in animal models, providing additional evidence that regular CVE training does efficiently amplify the antioxidant system and reduce oxidative stress and inflammatory responses, promoting health-related benefits. Specifically, the recent work by Alghadir et al. [190] demonstrated that following 24 weeks of moderate-intensity CVE training, both redox status and inflammatory state were improved in healthy older adults. A significant elevation was noted in the activity of TAC while MDA, 8-OHdG and high sensitivity C-reactive protein (hs-CRP) levels reduced substantially [190]. Also, 16 weeks of intense CVE training (80–85% of HRmax) induced a remarkable decrease in F2-isoprostane levels, in sedentary young women with the highest quartile of plasma F2-isoprostanes at baseline [191]. Moreover, swimming has been suggested to be a training mode that plays an important role in coronary vascular reactivity and the expression of antioxidant enzymes, justifying the exercise-induced reduction in the risk of coronary heart disease in postmenopausal women [192].

In terms of overweight/obese individuals though, the available evidence on the role of regular CVE training is limited and contradicting. Specifically, Youssef et al. [193] demonstrated that a 3-month multivariate CVE programme prevented the exercise-induced lipid peroxidation and inflammation in overweight/obese girls, possibly via an improvement in body composition. In contrast, Kelly et al. [194] reported previously that 8 weeks of CVE training failed to induce any alteration in levels of adipokines (C-reactive protein, interleukin 6, tumor necrosis factor alpha, adiponectin, leptin, and resistin), and oxidative stress markers (8-isoprostane) in overweight children. Table 2 lists all studies that examined the effects of regular CVE on redox status.

## 6. Resistance Exercise and Oxidative Stress

RE induces various health-related benefits to individuals, especially to those experiencing diminished muscle mass and function [212]. Relevant literature reports that RE is a safe exercise mode for CVD patients that elicits favorable effects on many of the established risk factors for cardiovascular diseases, such as hypertension, diabetes mellitus, obesity, increased plasma lipids and endothelial dysfunction [213,214,215,216,217,218]. Nonetheless, cardiovascular risks may be also included in a RE training program. For instance, it has been proposed that high-intensity RE (≥ 70% of 1RM) may induce excessive rise of blood pressure leading to cardiac hypertrophy and increasing as such the mortality risk [219,220]. Thus, it is recommended that a preliminary clinical examination is always performed in moderate- or high-risk cardiac patients prior to participation in RE training and in each training session, patients are closely supervised [218].

Despite numerous studies have investigated the effect of CVE on oxidative stress and inflammatory responses in CVD-related conditions, only a few researchers have examined the impact of RE on redox status and inflammatory profile of CVD patients. Research based on healthy population has revealed that a single bout of intense or unaccustomed RE induces an acute and transient inflammatory response and oxidative stress, whereas regular RE training increases the activity of anti-inflammatory mediators and enhances the antioxidant capacity [172,195,221,222]. This discrepancy among acute and regular RE is primarily attributed to the dual role of ROS in exercise, that function as damaging molecules following a single bout of exercise and as second messengers for cellular signal transduction pathways during prolonged exercise training, promoting the adaptation of skeletal muscle to training stimulus and enhancing the cellular responsiveness to subsequent exercise-mediated stress [223]. Recent evidence suggests that the protective effect of regular RE against oxidative stress is mediated via the increased expression of the transcription factors NF-κB and nuclear factor erythroid 2-related factor 2 (Nrf2) [223,224,225]. As reviewed in the work by Di Meo et al. [223], NF-κB is activated in response to exercise-induced ROS generation and translocate into the nucleus where it promotes transcriptional activation of antioxidant enzymes such as MnSOD, γ-glutamylcysteine synthetase (GCS) and catalase. Indeed, a recent study confirms this theory by providing evidence that following a 12-weeek RE training program, both older and young women demonstrated enhanced total antioxidant capacity and reduced malondialdehyde levels, accompanied by increased NF-κB stimulation [225].

Nrf2 is a redox-sensitive transcriptional factor that promotes adaptations to exercise by controlling the cellular antioxidant defense [223,226]. Upon its activation, Nrf2 regulates the expression of numerous antioxidant enzymes such as SOD, catalase and haem oxygenase-1, providing a strong cytoprotective effect [223,226]. It is activated in response to oxidative stress triggered by exercise and moves into the nucleus where it heterodimerizes with musculoaponeurotic fibrosarcoma proteins (Maf), binds to the antioxidant response element (ARE) and begins its transcriptional activity [223,224]. Alternatively, Nrf2 may be activated following exercise-mediated stimulation of the PI3K/Akt signaling pathway that induces depolymerization of actin allowing the formation of a complex with Nrf2 and finally promotes the nuclear translocation of the latter [224]. Although the current number of studies investigating the Nrf2 response to RE training is limited, RE is considered a potential inducer of Nrf2 signaling because of its well-established ability to increase the production of ROS as well as the stimulation of the PI3K/Akt signaling pathway [224]. In addition, a recent study conducted in chronic kidney disease patients on hemodialysis, reported that Nrf2 expression and GPx activity were both significantly increased following 3 months of RE [227], supporting further this hypothesis. On the following paragraphs we provide a thorough review of the available evidence related to acute and regular RE-mediated effects on oxidative stress.

### 6.1. Acute Resistance Exercise

Following an acute RE bout incorporating 9 sets of 10 repetitions at 75% of 1RM, both lipid peroxidation-related products and antioxidant concentrations were elevated in healthy men [173]. Also, the work by Atashak et al. [228] revealed that high-intensity RE induces oxidative stress, inflammation and cellular damage in athletes, that is in agreement with a previous report indicating that acute RE resulted in increased plasma levels of oxidative stress, in trained men [174]. Cardoso et al. [172] provided evidence that an acute RE session upregulates systemic indices of oxidative stress and reduces the antioxidant capacity in elderly women, immediately after the exercise. However, it seems that an age-related effect may exists, as a previous study showed a significantly higher activation of SOD in young compared to older adults, in response to an acute exercise bout [229]. Thus, it has been proposed that signal transduction of acute exercise may be impaired with aging [229].

Unlike to these findings, the notion that acute RE does not promote oxidative stress has been also reported. Specifically, a recent study showed that acute RE is safe for low-risk patients with coronary artery disease when performed at a low-to-moderate intensity (50–75% of 1 RM) without exacerbating the inflammatory status associated with their disease [213]. Moreover, Mcanulty et al. [230] reported that an exhaustive RE bout did not result in increased oxidative stress, as it was indicated by F2-isoprostane levels. Table 1 presents all studies that examined the effects of acute RE on redox status.

### 6.2. Regular Resistance Exercise Training

Regular RE offers multiple health-related benefits by promoting skeletal muscle mass gain, increased insulin sensitivity and blood glucose reduction, while it may also contribute to prevention and/or treatment of pathological states that affect metabolism and cardiovascular function [231]. In addition, regular RE has been reported to enhance the antioxidant capacity and prevent oxidative damage.

Sepifically, Li et al. [61] found that following 12 weeks of progressive RE training, phosphorylation of AKT and eNOS as well as expression of redox factor 1 and MnSOD were significantly elevated while FOXO1 phosphorylation decreased in rat aorta, providing evidence that RE can improve the function of the aorta and preserve redox status. By utilizing a rat model, Camiletti-Moiron et al. [232,233] also noted an antioxidant effect of RE, reporting increased catalase activity following a 12-week intervention with high-intensity RE training, despite no alteration was observed for the other antioxidant enzymes measured, such as MnSOD and Cu/ZnSOD.

In agreement with animal-based evidence, human studies have also confirm the protective role of RE against oxidative stress, cell damage and metabolism. Tarnopolsky et al. [222] reported RE training is an effective countermeasure for age-associated muscle atrophy, that is also associated with less oxidative stress and increased mitochondrial activity. In a previous work by Vincent et al. [195], six months of RE training resulted in reduced oxidative stress levels and homocysteine in both overweight/obese and normal-weight elderly adults. Likewise, a 12-week training period of low-intensity RE, substantially increased the amount of antioxidant enzymes and suppressed oxidative stress, in elderly men [196], while RE training over a 6-month period has been shown to reduce the exercise-induced oxidative stress and homocysteine regardless of adiposity, indicating that protection can be afforded in older, overweight/obese individuals as effectively as in healthy older adults [195]. More recently, it was reported that RE trained women exhibited improved antioxidant capacity and lower oxidative damage compared to their sedentary counterparts [172], supporting further the beneficial role of regular RE.

However, studies looking at the effects of training overload characteristics have revealed that training intensity, volume and duration differentially affect the RE-mediated responses. For example, Cakir-Atabek H et al. [197] suggested that RE training has a protective effect against oxidative stress, similar to that induced by CVE training, which is independent of training intensity. Likewise, RE training performed at a very low intensity (30–40% of 1 RM) has been shown to be unable to up-regulate the antioxidant defense system [175], supporting further the notion that high intensity is required for RE-mediated beneficial adaptations to occur. In contrast, findings from the work by Croymans et al. [234] revealed that high-intensity RE for 12 weeks, during which both training intensity and volume were progressively increased, did not alter blood lipid profile, total cholesterol levels, HDL, LDL, triglycerides and oxLDL.

Recently Carteri et al. [235] reported that RE protocols incorporating a single set of seven exercises, regardless of the exercise intensity or total workload, did not affect levels of oxidative stress in trained male individuals, suggesting that training volume is vital for the RE-mediated regulation of oxidative stress. This notion was later confirmed by the work of Flack et al. [198], who showed that 12 weeks of low-volume RE (7 exercises × 8-12reps) neither increased the oxidative capacity in skeletal muscle nor reduced mitochondria ROS production, in healthy older males. Furthermore, RE training interventions of the same intensity and volume but different duration induced dissimilar adaptations in a rat model [199]. Actually, the authors noted that GPx activity was significantly elevated and MDA levels were lower in heart tissue samples of rats performed 16 weeks of RE as compared to those performed only 4 weeks.

Thus, the available evidence strongly suggest that participation in regular RE activity, reduces levels of oxidative stress and the concomitant oxidative damage via an enhanced antioxidant defense mechanism. However, it seems that adequate training intensity, volume and duration should be incorporated in the training program to ensure that beneficial effect will occur. Table 2 presents all studies that examined the effects of regular RE on redox status.

## 7. Combined CVE and RE Training and Oxidative Stress

Although these two types of exercise training have been studied extensively and their combination has been recently documented to promote health-related benefits in a wide range of populations [236], the effect of combined CVE and RE training on oxidative stress has been minimally studied.

Several studies have provided evidence that CVE and RE induce similar effects on oxidative stress parameters [172,197,237]. Indeed, Azizbeigi et al. [200] demonstrated that endurance training, RE training and the combination of them induced the same changes in antioxidant capacity and systemic indices of oxidative stress. More recently, Soares et al. [201] showed that oxidative damage to DNA decreased while physical fitness and total antioxidant capacity increased in healthy men following 16 weeks of combined CVE and RE training. Moreover, 12 months of supervised, moderate-intensity, combined CVE, RE and flexibility training (total 140–270 min/ week, gradually increased) enhanced insulin sensitivity and cardiorespiratory fitness, reduced blood levels of LDL and contributed to the amelioration of oxidative stress in type 2 diabetes mellitus patients [202].

Therefore, despite limited, the available evidence consistently indicate that combined CVE and RE training has a strong potential to offer advantages against oxidative stress, particularly in conditions characterized by an upregulated pro-oxidant environment and redox balance disturbances. However, future research is required to further elucidate the beneficial role of combined CVE and RE training on oxidative stress and its application in clinical conditions, such as CVDs. The available studies that examined the effects of acute and regular combined CVE and RE exercise on redox status are presented in Table 1 and Table 2, respectively.

## 8. Exhaustive/Strenuous Exercise and Oxidative stress

Most of the studies reviewed here (Table 1 and Table 2) revealed that moderate-intensity CVE, RE or combined exercise training induce beneficial effects on oxidative stress markers. However, conflicting results have been also reported since oxidative stress markers were found to be decreased [238], increased [239,240,241] or remain unchanged [242] in response to an exercise stimulus. Factors such as the variability in animal or human characteristics, their training status, the antioxidant capacity, the type of diet, the variation in timing of tissue sampling, the differences in exercise intensity and duration, as well as methodological limitations, may either independently or collectively account for the discrepancy between these studies.

It appears though, that the exercise intensity is a crucial regulator of the oxidative stress response to exercise, as regular, low-intensity exercise decreases oxidative stress levels and encompasses increase antioxidant defense [243,244], whereas high-intensity or exhaustive exercise has been shown to increase free radical production and oxidative stress, reduce the antioxidant capacity and subsequently upregulates oxidative damage [245,246,247]. In particular, prolonged high-intensity exercise, such as marathon running, induces oxidative damage to proteins, lipids and DNA and could elicit deleterious effects on cardiovascular health [248,249]. Thus, strenuous or high-intensity exercise and regular, moderate-intensity exercise should be investigated separately and according to the type/mode of exercise performed (CVE vs. RE).

An early report noted that strenuous exercise increases free radical in skeletal muscle, causes oxidation of gloutathione and releases cytosolic enzymes and other sings of cell damage [245]. Numerous studies later, confirm these findings, providing further evidence that strenuous exercise increases the production of ROS and the neutrophil-mediated oxidative stress, promotes the release of transition metals while it also increases the interaction of methemoglobin with lipid peroxides and the activity of xanthine oxidase [246]. According to Gomez-Cabrera et al. [250] xanthine oxidase is involved in the generation of superoxide in response to exhaustive exercise. In addition, Popovic et al. [251] suggested that exercise to exhaustion induces the generation of oxidative stress predominantly by oxidative modification of protein molecules.

Recently, Sugama et al. [252] examined the oxidative stress and inflammatory response to an endurance race in two groups, of which one was characterized by increased muscle damaged and the other by only minor damage. In the highly damaged group, the authors observed that inflammatory markers, serum concentrations of diacron reactive oxygen metabolites (d-ROMs), but also anti-inflammatory markers, biological antioxidant potential (BAP) and TAC tended to be higher compared to those in the minor damaged group, immediately after the race.

Moreover, Jorde et al. [253] presented evidence that after exercise to exhaustion, oxidized oxLDL was increased in CHF patients but not in their healthy counterparts. On the other hand, Goff and colleagues [254] reported that although oxidative stress indices are substantially increased following strenuous exercise, cardiac biomarkers remain unaffected, concluding that strenuous exercise may be performed without any risk by patient or individuals at high risk of heart disease. Thus, although the available evidence strongly suggest that strenuous exercise or exercise to exhaustion is highly damaging and induces oxidative stress to a much greater extent than exercise of moderate or low intensity, the clinical perspective of strenuous exercise warrants further investigation to enhance our understanding of the exercise prescription in clinical populations.

## 9. Detraining and Oxidative Stress in CVDs

The term ″detraining‶ refers to complete or partial loss of training-induced adaptations, in response to absence of or insufficient training stimulus [255]. According to Toraman and Ayceman [256] the detraining effects are age-dependent, while clinical conditions such as CVD may also affect the training-mediated adaptations. Notably, it has been proposed that the effect of detraining on CVDs and oxidative stress is equally important with that of exercise training, as it regulates the time-frame during which the cardiovascular-related adaptations will be diminished [257]. Thus, a question may arise as to whether the type/mode and intensity of the exercise or the duration of it affect mostly the detraining effects.

### 9.1. Resistance Exercise Training and Detraining

Thus far, the effect of detraining on the RE training-induced adaptations has been mostly examined in healthy young and older individuals. Specifically, Miyachi et al. [258] showed that improvements in LV wall thickness, LV mass index, LV hypertrophy index and arterial compliance values induced by a 4-month high-intensity RE training (80% of 1RM), returned to baseline values after 4 months of detraining. The authors concluded that a detraining period of 4 months is sufficient to completely reverse the cardio-protective effect mediated by RE training. Likewise, Stebbings et al. [259] reported that a short detraining period of 2-4 weeks after 8 weeks of progressive, lower limb RE training (80% of 1-RM), resulted in a complete reversal of the adaptions observed in muscular strength, resting heart rate, superficial femoral *artery* (*SFA*), carotid *artery* (*CA*) diameters and mean blood flow [259]. Notably, most of these maladaptations occurred within the first 2 weeks of detraining, with reductions below baseline levels being apparent in SFA diameter and CA blood flow [259], indicating that changes in resting arterial diameter and blood flow occur rapidly in the absence of exercise training (detraining).

However, more recent reports have consistently shown that muscular strength gains following resistance training of moderate-to-high intensity, can be maintained above baseline levels for as long as 2 to 52 weeks of detraining, in older individuals [260,261,262,263,264,265]. Specifically, Harris et al. [264] noted that despite strength losses, significant levels of total-body strength were retained even after 20 weeks of detraining following an 18-week progressive RE training. In addition, despite a significant reduction was observed in muscle strength/size after 12 weeks of detraining, the participants’ (older women) muscle strength/size was still significantly higher compared to baseline values [263]. In that study 12 weeks of home-based moderate-intensity RE training using low-load elastic bands (with blood flow-restricted) preceding the detraining period. In agreement with these findings, Coetsee and Terblanche [262] demonstrated that gains in muscle strength and submaximal endurance capacity were not completely lost after 16 weeks of detraining in older individuals who has previously participated in a 16-week, progressive RE training programme.

The work by Fatouros et al. [265] revealed that training intensity is a crucial factor for both training adaptations and detraining-induced maladaptations. Actually, the authors showed that 24 weeks of high-intensity RE training induced greater gains in strength, anaerobic power and functional capacity compared to 24 weeks of low-intensity RE training, in elderly individuals [265]. Interestingly, following 48 weeks of detraining, it was observed that the high-intensity RE training protocol was more effective than low-intensity RE training at maintaining the exercise-induced adaptations [265]. Therefore, it appears that exercising at a higher intensity not only results in greater strength gains but also in a lower rate of strength loss during detraining, maintaining increased strength and functional performance for a longer period of time.

Data specific to CVD patients are currently lacking. The only study that examined hypertensive women, reported that improvements on blood pressure and muscular strength induced by RE training at a moderate intensity over a 14-week period were preserved following 14 weeks of detraining [266]. Likewise, there are no published reports on the effect of detraining on oxidative stress adaptation to RE training in the context of CVDs. A recent study showed that the improvement in antioxidant capacity induced by a 12-week RE training, as evident by an increase in total radical-trapping antioxidant parameter, was preserved following a subsequent detraining period for 12 weeks, but this study was also performed in healthy older women [267]. Thus, it is evident that future studies are required to shed light on the effects of detraining on RE training-induced adaptations of oxidative stress, antioxidant capacity as well as physiological and performance-related parameters, in CVD patients.

### 9.2. Cardiovascular Exercise Training and Detraining

Animal-based studies investigating the role of detraining in adipose tissue have pointed out that detraining might play a role as a possible obesogenic factor, increasing glucose uptake and oxidation [268,269,270]. Specifically, 8 weeks of moderate-intensity CVE training reduced the adipocyte size in rats, while a subsequent 4-week detraining period caused adipocyte hypertrophy, either accelerated their weight gain rate or completely recovered it, and also allowed total adiposity to recover by increasing the lipogenic capacity [268]. The authors suggested that detraining might have stimulated the adipogenic process and attenuated apoptotic events which contribute to a rapidly recover of the adipose mass [268]. A more recent work by the same group [269] revealed that physical training (8-weeks, moderate-intensity CVE) interruption did not result in rapid loss of the acquired adaptations. In that study, during a 4-week detraining period, the adipocytes from detrained rats were more responsive to insulin and more effective in taking up glucose when stimulated with insulin compared to those from the sedentary group [269]. Based on this evidence, the authors hypothesized that, the increased glucose entrance in the adipose tissue cells from detrained rats resulted in increased substrate availability for triacylglycerol synthesis, while the accelerated oxidation of glucose enhanced the energy provision for triacylglycerol synthesis.

Furthermore, it has been proposed that anatomical and functional adaptations gained throughout a 4-week CVE training (80–85% VO_2_max) are lost in rats within 4 weeks of detraining [271]. Accordingly, Kemi et al. [257] reported that training-induced improvements in VO_2_max and myocardial responses reversed within 3–4 weeks of detraining while the endothelium-related adaptations were completely abolished at 2 weeks. Moreover, a 2-week detraining period following 10 weeks of moderate-intensity CVE training, resulted in reduced VO_2_max by 22% and reversed resting heart rate near baseline values, in Wistar rats [272].

Another investigation, focusing on the effects of training and detraining on functional and inflammatory responses in CVDs, have pointed out that improvements in autonomic modulation of heart and vessels as well as reduction in inflammatory cytokines induced by a moderate-intensity CVE training (2 months) in rats with myocardial infraction, were sustained even after one month of detraining [273]. In contrast, Agarwal et al. [98] showed that the exercise-induced improvements in pro-inflammatory cytokines, cardiac hypertrophy and diastolic function as well as the attenuation of mitogen-activated protein (MAP) expression were not affected by a 2-week detraining, preceded by 4 weeks of moderate-intensity CVE training in AngII-induced hypertensive rats, but detraining abolished the exercise-induced attenuation of oxidative stress and improvement in IL-10 within the paraventricular nucleus (PVN) of these rats.

Similar responses to detraining have been also observed in human studies. At the beginning of 2000, Maeda et al. [274] showed that the CVE training-mediated (8-weeks of moderate intensity) increase in nitric oxide level (vasodilator substance) and the decrease in endothelin-1 level (vasoconstrictor peptide) in healthy individuals lasted up to the 4th week of detraining and returned to the pre-exercise levels in the 8th week. Furthermore, Koshiba and Maeshima [275] reported that diastolic pressure and changes in arterial stiffness are maintained after a detraining period of 6 and 3 months, respectively, in endurance athletes. These findings are in agreement with the results of a previous study [276], showing that 6 months of intensive CVE training (progressive) in young, untrained subjects lead to increased aerobic fitness, LV mass, LV end-diastolic volume (LVEDV), and interventricular wall thickness, whereas no changes observed in these parameters in RE training (65–85% of 1-RM) group. In that study, all changes had returned to baseline values after 6 weeks of detraining, except LVEDV that remained elevated [276]. In addition, Toraman et al. [261] reported that six weeks of detraining failed to reverse the gains in aerobic endurance and agility adopted during a preceding 9-week training programme, as well as the gains in lower body strength in older adults (aged 60–86 years). However, when a more prolonged detraining (52 weeks) was applied, a complete loss of all gains adopted during the 9-week training programme and a dramatic decrease in aerobic endurance were observed, in subjects aged > 74 years [261].

Collectively, these data provide strong evidence that detraining elicits ″deleterious‶ effects on CVE training-induced adaptations by promoting maladaptations related to physiological parameters, metabolic processes, inflammation and oxidative stress. However, more studies are required in the context of CVDs, to improve our understanding of the impact that detraining has on the CVET-mediated cardio-protective effects.

### 9.3. Combined Exercise Training and Detraining

The available studies examining the impact of detraining on adaptations induced by combined exercise have mainly focused on blood lipid profile. Actually, there has been shown that an 8-month training programme combining CVE and RE at a moderate intensity, induced favorable adaptations on total cholesterol, triglycerides, HDL cholesterol (HDL-C) and apolipoproteins A1 (apo-A1) levels, in patients with coronary artery disease [277]. However, the authors reported that within the subsequent 3-month detraining period, all adaptations were fully reversed [277]. A recent study investigated the effects of a 3-month detraining period on muscle strength and blood lipid profile, following an 8-month training programme (CVE vs. RE vs. combined) in individuals with coronary artery disease [278]. Interestingly, after 3 months of detraining, muscle strength was still significantly higher compared to the baseline values in both RE and combined exercise training groups but not for the CVE group [278]. Similar results were observed for TC, TG, HDL-C and LDL-C that were still significantly lower compared to baseline values at the second month of detraining for the CVE and combined exercise training groups but not for the RE training group [278]. Furthermore, all groups revealed favorable alterations in hs-CRP lasted until the 1st month of detraining [278].

In addition, Yuing Farias et al. [279] provided evidence that combined moderate-intensity CVE and RE training protocols for 6 weeks are able to improve lipid profile, glycaemia in a fasted state and the level of HbA1C in a group of non-medicated individuals with type 2 diabetes Mellitus. After 6 weeks of detraining though, the RE training protocol was shown to be more effective at maintaining the exercise-induced adaptations on HDL-C, LDL-C and HbA_1C_ compared to CVE training.

## 10. Physical Inactivity and Oxidative Stress in Cardiovascular Diseases

It has been proposed that, physical inactivity leads to impairment of physiological functions and reduces the whole body resistance to oxidative stress [280]. Indeed, oxidative stress levels have been consistently shown to be considerably higher in sedentary compared to physically active adults [170,203]. Accordingly, Radak et al. [281] showed that levels of 8-Oxoguanine (8-oxoG) in old inactive individuals are higher compared to their active counterparts, indicating that regular physical activity promotes an adaptive response that involves a more efficient antioxidant defense mechanism and DNA repair machinery. Interestingly, elderly inactive men exhibit significantly higher oxLDL and lower total plasma antioxidant status (TAS) compared to either young inactive or elderly active group [32]. In a recent work by Park and Kwak [176], there was no difference in resting oxidative stress levels and antioxidant capacity between untrained and trained (RE and endurance trained athletes) young men, however, the untrained ones exhibited significantly higher MDA and PC levels following an acute bout of exercise compared to trained ones.

Furthermore, the physical inactivity-mediated reduction in antioxidant capacity and enhancement of oxidative stress is detrimental for the overall health status as it has been associated with increased incidence of oxidative stress-related diseases [64,203]. Specifically, it was demonstrated that physical inactivity had a positive association with oxidative stress and cardiovascular risk factor such as blood pressure, BMI and percent (%) body fat, in postmenopausal women [102], while a previous study in mice showed that physical inactivity increases vascular oxidative stress, ROS production and protein expression of the NADPH oxidase subunits p47phox and p67phox in the endothelium and media of the aortic wall, develops atherosclerotic lesions in the aortic root and ascending aorta, and finally impairs endothelium-dependent vasorelaxation of inactive mice as opposed to more active animals [147].

Collectively, these findings provide strong evidence that sedentary lifestyle is associated with enhanced vascular oxidative stress and reduced antioxidant capacity, which, in turn, propagate chronic oxidative stress-related diseases. Also, alterations in inflammation and oxidative stress are more prominent with advancing age and age-related diseases, such CVD.

## 11. Conclusions

In contrast to antioxidant supplements, regular exercise has been shown to elicit more favorable effects on physical function and resistance to oxidative stress, and thus, it has attracted an increasing interest from researchers focusing on prevention and treatment of CVDs. Exercise training represents a powerful signal for molecular events leading to activation of signal transduction pathways that promote activation of antioxidant enzymes, anti-inflammatory mediators as well as upregulation of proteasome activity. Consequently, it results in reduced oxidative stress levels, enhanced antioxidant capacity preserving as such redox balance and cellular homeostasis. Acute CVE results in a substantial elevation of oxidative stress-related indices during recovery that is proportional to exercise intensity and individual’s physical conditioning status. However, when regularly performed, CVE amplifies the endogenous antioxidant enzyme activity and reduces levels of oxidative stress in an intensity-dependent manner, as early as within 4 to 6 weeks of training. Acute RE on the other hand, increases oxidative stress, inflammation and cellular damage both in young trained and elderly individuals when performed at a high intensity, whereas it is likely that these effects are mitigated when low-to-moderate intensity RE is performed. Similarly to CVE, regular RE enhances the antioxidant capacity, reduces oxidative stress and preserves redox status when adequate training intensity and volume are incorporated in a training program that lasts at least 4 weeks. Finally, it is evident that detraining, following either RE or CVE training, induces maladaptations that are inversely related to training intensity. Also, it appears that RE training is more effective at maintaining the exercise-induced adaptations during detraining, as compared to CVE training.

## Figures and Tables

**Table 1:** Effects of Acute Exercise on Redox Status.

Exercise Type	Study Subjects	Training Protocol	Findings	Reference
CVE	Young males (*n* = 16)	High intensity interval test on cycloergometer: 8 bouts of 1 min at 100% of peak power Blood samples: pre, immediately post, 30 min post exercise	↑ TBARS 30 min post exercise ↔ GSH, SOD ↓ CAT 30 min post exercise ↓ Lymphocyte proliferative	Gomes et al., 2016 [109]
CVE	Healthy males (*n* = 10)	CrossFit protocol: 5 pull-ups, 10 push-ups, 15 air-squats in 20 min (as many rounds as possible) High intensity treadmill protocol: Running (90% HRmax) for 20 min	immediately post, 1-h and 2-h post exercise Oxidative stress markers: ↑ LOOH ↓ PC Antioxidant capacity markers: ↑ FRAP ↓ TEAC	Kliszczewicz et al., 2015 [165]
CVE	Diabetes patients and healthy group	A single bout of a 3 h walk, 30% heart rate reserve	↔ Oxidative stress in both groups ↑ anti-oxidant defense	Francescato et al., 2014 [166]
CVE (swimming)	Male Wistar rats (6 groups): 30 Control group (C): sedentary G3: below anaerobic threshold G4: below an. threshold. G5: an. threshold. G6: an. threshold. G8: above an. threshold.	1-h swimming carrying metal ring: 3% (G3), 4% (G4), 5% (G5), 6% (G6), 8% (G8) of their body weight	↑ lipid peroxidation (MDA) in trachea and lung in all exercise groups G3: ↑ MDA in rat trachea G4: ↑ MDA in rat trachea G5: ↑ MDA in rat trachea G6: ↑↑ MDA in rat trachea G8: ↑↑↑ MDA in rat trachea	Brito et al., 2015 [168]
CVE (swimming)	Male Wistar rats (6 groups): 30 Control group (C): sedentary G3: below anaerobic threshold G4: below an. threshold. G5: an. threshold. G6: an. threshold. G8: above an. threshold.	1-h swimming carrying metal ring: 3% (G3), 4% (G4), 5% (G5), 6% (G6), 8% (G8) of their body weight	↑ MDA in heart and aorta in all exercise groups G3: ↑ MDA in aorta & heart G4: ↑ MDA in aorta & heart G5: ↑ MDA in aorta & heart G6: ↑↑ MDA in aorta & ↑ MDA in heart G8: ↑↑↑ MDA in aorta & heart	Brito et al., 2015 [169]
CVE	Young males (*n* = 30, age = 21 ± 2, 3 groups) WT = Well-trained group (*n* = 10) MT = moderate trained group (*n* = 10) UT = untrained group (*n* = 10)	All groups performed an acute bout of aerobic exercise: 5 min running with 50% VO_2_max & 30 min running 70% VO_2_max Blood samples: pre, immediately post, 10 min post and 30 min post exercise protocol.	↑ GSH in MT compared with UT & WT groups ↓ GSSG in MT compared with UT & WT groups ↑ GSH/GSSG MT compared with UT & WT groups ↑Cortisol and CK after exercise in all groups	Seifi-Skishahr et al., 2016 [171]
CVE and RE	Women (45–55 y) Resistance group (RE): Followed 2 y resistance training program Aerobic group (AE): Followed 2 y aerobic training program Control group (C): sedentary women	Acute bout of exercise: RE: 10 rep. ~75–80% of 1RM × 10 stations AE: 50 min on cycle ergometer ~75–80% of HR C: No exercise Blood samples: pre, post, 1h post exercise	Rest: Levels of SOD and CAT in RE and AE > C group. Post exercise: ↓ SOD and CAT in RE and AE. ↑ TBARS & protein carbonyls 1 h post exercise: ↓ SOD and CAT in RE and AE. Oxidative stress: TBARS return at baseline levels, protein oxidation remains elevated	Cardoso et al., 2012 [172]
RE	Men	10 exercises × 9 rep. ~75% of 1 RM Blood samples: 30 min pre, immediately post exercise	↑ Lipid oxidation ↑ antioxidant concentrations	Ramel et al., 2004 [173]
RE	Trained men	Acute bout of exercise: 3 × 10 rep. ~75% of 1 RM 90 s rest between sets	↑ TBARS (42%), AOPP (28%), uric acid (27%) and GSH (14%), uric acid (36%)	Deminice et al., 2010 [174]
RE	Males (*n* = 16, age = 25 ± 4, 2 groups) Untrained group (UT, *n* = 8) Resistance trained group (RT, *n* = 8)	Both groups performed one acute bout of a progressive RT protocol (leg extension): 1 × 17 reps at 50% of 1 RM, 1 × 14 reps at 60% of 1 RM, 1 × 12 reps at 70% of 1 RM, 2 × 5 reps at 80% of 1 RM, 3 × 3 reps at 90% of 1 RM, 5 min rest between each intensities, 90–120 sec. rest between sets. Blood collection: pre, immediately after each intensity, 30 min post, 60 min post, 24 h post exercise bout	↑ Blood lactate → parallel with the rise of ex. intensity in both groups. ↑ PC during ex. bout and approached the baseline values in recovery period, in both groups ↔ Serum glutathione (GSH) ↑ SOD during ex. and 30 min post ↑ Lipid peroxidation (LHP) and approached the baseline values in recovery period, in both groups	Cakir-Atabeck et al., 2015 [175]
CVE (test)	Healthy young males (3 groups): Competitive endurance athletes (ET) Resistance trained athletes (RT) Untrained individuals (UT)	Grated exercise test: Treadmill peak oxygen consumption test. Starting with 3% elevation for 3 min and increasing 1.5% per min until exhaustion.	Oxidative stress markers MDA and PC: ↑ in UT group ↔ in ET and RT groups Antioxidant markers TAC: ↓ in ET and RT groups ↓↓ in UT group	Park & Kwak, 2016 [176]
RE	Chronic kidney patients (*n* = 16)	Four Strength exercises in both lower limbs with ankle –cuffs and elastic bands (60% of 1-RM) 3 sets × 10 rep, rest: 3 min between each exercise and 1 min between each set.	↓ SOD after acute exercise ↔ CAT, GPx, MDA and hs-CRP levels	Esgalhado et al., 2015 [177]
CVE	Sedentary group (7 males & 8 females: age 65.8 ± 3.3 y.) (score < 9 on the questionnaire of physical activity) corresponds to a sedentary life style. Active group (8 males & 10 females: age 65.1 ± 3.5 y.) (score 9–16 on the questionnaire of physical activity) corresponds to an active life style.	Low intensity aerobic exercise: (a) 5–10 min warm-up (b) 15–20 min aerobic exercises (walking, dancing, and aerobics) (c) circuit muscular endurance exercise with elastic bands and free weights (knee flexion, arm raise, shoulder abduction, shoulder rotation, squatting, biceps curl etc.) rest 60–120 sec.	At Rest: (1) SOD levels for the active group > sedentary group. (2) No differences between groups in α-Tocopherol, GR, MDA and GPX. 20 min post-exercise: (1) SOD levels for the active group > sedentary group (2) α-Tocopherol: ↔ in sedentary group, ↑ in active group. (3) GR: ↔ in both groups. (4) MDA: ↑ in both groups. (5) GPX: ↔ in sedentary group, ↑ in active group.	Bouzid et al., 2014 [178]
CVE	Women	A single bout of 30 min run, 70% VO2 max	↑ Lipid hydroperoxides, protein carbonyls, GSH, GSSG, TNF-a & interleukin-6	McKenzie et al., 2014 [179]
CVE	Trained men	A single bout of: (a) 60 min run, 70% HR reserve, (b) 5 × 60 sec. sprints, 100% max capacity,(c) 10 × 15 sec. sprints, 200% max capacity (d) No exercise rest	↔ malondialdehyde, hydrogen peroxide, advance oxidation protein products. ↔ trolox equivalent antioxidant capacity, superoxide dismutase, catalase, glutathione peroxidase.	Canale et al., 2014 [180]
RE	Trained men	Acute bout of exercise: 7 × 4. 60–90% of 1RM	↑ urinary 8-OHdG excretion and plasma MDA levels	Rahimi, 2011 [181]
CVE	Sedentary group (4 males & 8 females) Active group (5 males & 8 females)	A single bout of 30 min run Sedentary: ~55% VO_2_max Active: ~70% VO_2_max	↑ oxidative stress (↓ lag time LDL oxidation) ↑ plasma MOP protein	Wetzstein et al., 1998 [182]

8-OHdG: 8-hydroxy-2′-deoxyguanosine; AOPP: oxidation protein products; CAT: catalase; CK: creatine kinase; CVE: cardiovascular exercise; FRAP: ferric-reducing antioxidant power; GPx: glutathione peroxidase; GR: glutathione reductase; GSH: reduced glutathione; GSSG: oxidized Glutathione; HRmax: maximum Heart Rate; hs-CRP: high-sensitivity C-reactive protein; LA: Blood lactate; LDL: low-density lipoprotein; LHP: Lipid peroxidation; LOOHs: lipid hydroperoxides; MDA: malondialdehyde; MOP: myeloperoxidase; PC: protein carbonyls; RE: resistance exercise; RM: repetition maximum; SOD: superoxide dismutase; TAC: total antioxidant capacity; TBARS: thiobarbituric acid-reactive substances; TEAC: trolox-equivalent antioxidant capacity; ↑: significant increase *p* < 0.05 vs. control; ↑↑: significant increase *p* < 0.05 vs. control; *p* < 0.05 vs. 4%; ↑↑↑: significant increase *p* < 0.05 vs. control; *p* < 0.05 vs. 4%; *p* < 0.05 vs 6%; ↓: significant decrease vs. pre; ↓↓: significant decrease vs. pre, ET and RT group; ↔: no significant change.

**Table 2:** Effects of Regular Exercise on Redox Status.

Exercise Type	Study Subjects	Training Protocol	Findings	Reference
RE training (12 weeks)	Male F344 rats (*n* = 12, 2 groups) Sedentary/control (C, *n* = 6) Climbing exercise group (RT, *n* = 6)	Progressive RE protocol: Climbing a ladder 135 cm length (grid step 2.5 cm, grade 60 degree) Weight load attached to their tails. 1st circle 50% of their body weight (Bw) → 2 min rest 2nd circle 75% of their Bw → 2 min rest 3rd circle 90% of their Bw → 2 min rest 4th circle 100% of their Bw → 2 min rest 5th circle 100% + 30 g of their Bw → 2 min rest Training was stopped when rats refused to climb.	Aortic rings under 40× and 200× magnification: no significant difference between groups. In the aorta of rats: ↑ eNOS and AKT phosphorylation in RT group ↑ MnSOD and Redox factor-1 in RT group ↓ FOXO1 phosphorylation in RT group	Li et al., 2015 [61]
CVE training (8 weeks)	Sprague Dawley rats (*n* = 60, 4 groups) Sh = Sham sedentary group Sh + ex = sham with exercise OVX = ovariectomized sedentary group OVX + ex = ovariectomized with exercise group	Exercise groups (Sh = ex, OVX + ex) performed CVE training: Running 15 min/day for the 1st week and 60 min/day at 18 m/min for 7 weeks.	Effects of exercise on: ↑ CSE expression in myocardium in OVX + ex group Anti-oxidative defense in myocardium: ↑ TAC in OVX + ex group CAT & SOD → not change in OVX + ex group ↓ CAT & SOD in sham + ex group Oxidative stress markers in myocardium: ↓ MDA level in OVX + ex group	Tang et al., 2016 [65]
RE training (6 weeks)	Healthy young individuals (*n* = 32, 2 groups): African Americans (AA, *n* = 14) Caucasian (Cau, *n* = 18)	Moderate RE training: 3 sessions/week, 60 min/session 2-way body part split: legs, back and biceps on one day; chest, shoulder and triceps on a separate day.	↑ Strength in both groups ↓ blood pressure in Cau ↔ blood pressure in AA ↓ MMP-9 in AA ↔ MMP-9 in Cau ↓ 8-isoprostane (8-IsoP) in AA ↔ IL-10, TNF-a, sVCAM-1, MMP-2	Cook et al., 2013 [91]
CVE training (12 weeks)	Spontaneously hypertensive rats	12 weeks, 5 days/week, 60 min/session, 55–65% max running speed	↓ oxidative stress ↑ NO bioavailability ↓ blood pressure Improve mechanical and functional alterations of the coronary and small mesenteric arteries	Roque et al., 2013 [94]
CVE training (4 weeks)	Wistar rats (*n* = 80, 8 groups) CS = control + sedentary, CE = control + exercise, CSQ = control + sedentary + quercetin, CEQ = control + exercise + quercetin, DS = diabetes + sedentary, DE = diabetes + exercise, DSQ = diabetes + sedentary + quercetin, DEQ = diabetes + exercise + quercetin.	CE, CEQ, DE & DEQ performed moderate chronic aerobic exercise (swimming) 1 h/day, 5 days/week.	↓ MDA & PC levels in aortic tissue in exercises group ↑ SOD & CAT in aortic tissue in exercises groups ↓ NOx levels in aortic tissue in exercises group ↓ iNOx levels in aortic tissue in exercises group	Chis et al., 2015 [183]
CVE training (9 weeks)	Male Wistar rats (4 groups): Sedentary Control (C, *n* = 14) Exercise control (C-Ex, *n* = 15) Sedentary diabetes (DM-C *n* = 25) Exercise diabetes (DM-Ex, *n* = 25)	Low intensity physical exercise training: Running duration 18 min/day, Speed 11 m/min, 5 days/week	Lipid hydroperoxide: in DM-C > C and DM-Ex SOD and Catalase: DM-Ex > DM-C > C Glutathione peroxidase: DM-C < C and DM-Ex	Gimenes et al., 2015 [184]
CVE training (8 weeks, Swimming)	Rats (4 groups): 20 Healthy rats sedentary(H): 5 Healthy + Exercise (H + Ex): 5 High fat Diet sedentary (HFD): 5 High fat Diet + EX (HFD + Ex): 5	H+Ex and HFD+Ex group: 1-h Moderate intensity swimming for 8 weeks	H group: ↔ MDA in heart tissue, LOX-1 protein → expressed in heart cells H + Ex group: ↔ MDA, ↓ gene expression of LOX-1 receptor HFD group: ↑MDA, ↑ gene expression of LOX-1 receptor HFD+Ex group: ↓ MDA, ↓ gene expression of LOX-1 receptor	Riahi et al., 2015 [185]
CVE training (8 weeks)	Adult rats	8 weeks, 5 days/week, 60 min/session, 60% max running speed	↑ running distance ↑ antioxidant defense system ↑ superoxide dismutase (SOD)	Coelho et al., 2013 [186]
CVE training (6 weeks)	Male Wistar rats (4 groups): 28 C = Control group (*n* = 7) EX = Exercise group (*n* = 7) D = Diabetes group(*n* = 7) EX + D = Exercise + Diabetes (*n* = 7)	Ex group and EX + D group: Free access to running wheel 24 h/day for 6 weeks	↓ MDA ↑ SOD, GPx, TAC	Naderi et al., 2015 [187]
CVE training (10 days)	Sprague-Dawley rats (2 groups): Sedentary (SED) Endurance training group (Ex)	Exercise group: moderate intensity treadmill training Running duration 60/day Intensity 30 m/min (70% max oxygen consumption)	24 h after the final training ↔ 4-hydroxynonenal conjugated proteins (4-HNE) in both groups ↑ SOD2 ↑ CAT	Holland et al., 2015 [188]
CVE training (24 weeks)	Healthy older individuals (*n* = 100, 2 groups) C = control group (*n* = 50) EX = exercise group (*n* = 50)	Moderate CVE: 45–60 min on treadmill, bicycle or Stair master, intensity 60–70% of HRmax, 3days/week.	↓ MDA & 8-OHdG ↑ TAC ↓ hs-CRP Significant correlation between oxidative stress markers and hs-CRP	Alghadir et al., 2016 [190]
CVE training (16 weeks)	Women	16 weeks, 5 days/week, 30 min/session, 80–85% HRmax	↔ Body weight & BMI, ↑ aerobic fitness ↓ systemic oxidative stress only in women with the highest quartile of plasma F2-isoprostanes at baseline (≥57 pg/mL)	Arikawa et al., 2013 [191]
RE training (24 weeks)	Untrained healthy individuals (*n* = 49, age = 60–72, 4 groups) Control normal weight group (no exercise, Cn) Control obese group (no exercise, Co) Exercise normal weight group (REN group) Exercise obese group (REO group)	REN & REO group performed moderate RE program: One set of 13 exercises × 8–13 reps (50–80% of 1RM) 3 days/week	↑ muscle strength, VO_2_max in REN & REO group ↔ total cholesterol and HDL-C ↓ Lipid hydroperoxides and TBARS (REN & REO < Cn & Co) Homocysteine in plasma: REN & REO < Cn & Co	Vincent et al., 2006 [195]
RE training (12 weeks)	elderly men	12 weeks, 3 sessions/week, 3 sets × 10 repetitions each of leg press and leg extension (50–80% 1 RM)	↑ muscle antioxidant capacity (82.5% catalase activity, 75% CuZnSOD activity)	Parise et al., 2005 [196]
RE training (6 weeks)	Young men	6 weeks, 3 days/week 2 groups: Hypertrophy-intensity group (3 × 12 rep. ~70% of 1 RM) strength-intensity group (six exercises of 3 sets × 6 rep. ~85% of 1 RM)	In both groups: ↓ MDA ↑ GSH	Cakir-Atabek et al., 2010 [197]
RE training (12 weeks)	Older adults (*n* = 19, age ≥ 60 years, 2 groups) Control group (C, *n* = 8) RT group (RT, *n* = 11)	RT group performed: Supervised RT 3 days/week 3 upper body exercises 4 lower body exercises 1 set × 8–12 reps each exercise to volitional fatigue Muscle biopsies: pre, 48 h post, after the last RE session at 3 & 12 weeks.	↑ Muscle strength ↔ Pyruvate oxidation, acid soluble metabolites and total fatty acid oxidation	Flack et al., 2016 [198]
RE training (4–16 weeks)	Wistar male rats (*n* = 10, 3 groups) Sedentary–Control (C group) Exercise-1 (4 weeks training, RE-1 group) Exercise-2 (16 weeks training, RE-2 group)	Regular RE in a squat training device cylinder 4 sets × 12 reps/day, 90 min rest between each set, 5 days/week	Heart tissue: ↑ GPX only in RE-2 group ↑ MDA only in RE-1 group SOD → no changes Cell damage enzymes: ↑ LDH & CK → only in RE-1 group	Ghiasi et al., 2015 [199]
CVE training RE training Combined training (CT) (8 weeks)	Untrained men 3 groups: CVE: *n* = 10 RE: *n* = 10 CT: *n* = 10	CVE: incremental running up to 80% of max HR RE: incremental RE beginning load 50% up to 80% of 1 RM CT: Combination CVE and RE every other day during the week	In all three training groups: ↑ SOD, erythrocyte GPx, TAC ↓ MDA No significant difference in the interaction of time and group between variables of SOD and GPx enzymes and TAC of plasma and MDA.	Azizbeigi et al., 2014 [200]
Combined exercise training (16 weeks)	Healthy men (40–74 years, 2 groups): C = control group (*n* = 26, no exercise, age: 52 ± 9) Ex = exercise group (*n* = 31, age: 58 ± 10)	Ex group performed moderate combine exercise training: 3 days/week, 60–75 min/session consisted of: CVE: 25–30 min/session (75% of HRR) RE:30–35 min/session (65–75% of 1 RM, 10–15 reps × 3 sets, bench press, leg press, leg curl, leg extension, latissimus, abdominals, arm flexion) Stretching & cool down: 5–10 min.	↓ MDA ↑ TAC ↓ DNA strand breaks ↓ oxidative DNA damage (FPG-sensitive sites) ↔ DNA repair capacity (8-oxoguanine DNA glycosylase)	Soares et al., 2015 [201]
CVE vs. RE vs. flexibility training (12 months)	Healthy Male subjects and with type 2 diabetes mellitus (3 groups): 30 Healthy group (H) Control group (CT2MD) Training group(ExT2MD)	ExT2MD group: moderate CVE (cycling progressively increase 15 min to 35 min per session), RE (major muscle groups × 3 sets × 12 rep) and flexibility (Static stretching) training (total 140–270 min/week, gradually increased)	ExT2MD group: ↓ oxPAPC compared with T2MD group, ↑ oxPAPC compared with Healthy group T2MD group: ↑↑ oxPAPC compared with Healthy group	Vinetti et al., 2015 [202]
CVE training	Postmenopausal women	Compared physical active with sedentary subjects, on oxidative stress markers.	↑ oxidative stress markers in sedentary versus active women	Bartfay, W. & Bartfay, E., 2014 [203]
CVE training	Elderly men	Compared physical active with sedentary subjects, on oxidative stress markers, after an incremental exercise test	Low intensity aerobic exercise prevent the decline of antioxidants linked with aging	Bouzid et al., 2014 [178]
CVE training (12 weeks)	Rheumatoid arthritis patients	3 months, 3 sessions/week, 30–40 min/session, 70% VO2 max	↔ Markers of oxidative stress ↓ 3-Nitrotyrosine ↓disease activity	Wadley et al., 2014 [204]
CVE training (16 weeks)	Obese & Type 2 Diabetes men	16 weeks, 3 sessions/week, 2 groups: a) low intensity (30–40% VO_2_max) b) moderate intensity (55–65% VO_2_max)	↔ Body composition and aerobic fitness Improve oxidative stress markers especially when performed moderate intensity protocol.	Krause et al., 2014 [205]
Combined CVE and RE training (6 weeks)	Women with metabolic syndrome	6 weeks, 3 sessions/week, 60 min/session CVE and RE	↓ indicators of oxidative stress, arterial pressure levels, pulse pressure and the Augmentation Index ↑ cardiovascular fitness	Eleuterio-Silva et al., 2013 [206]
RE training (8 weeks)	Men	Progressive RE-training 8 RE on nonconsecutive days for 8 weeks at 50% of 1RM and reached 80% 1RM by Week 8	↑ SOD ↓ MDA ↔ erythrocyte GPx & TAC levels	Azizbeigi et al., 2013 [207]
RE training (8 weeks)	Men	moderate (MR) and high resistance (HR) training	↑ SOD activity in MR (*p* = 0.026) and HR (*p* = 0.044) groups. ↑ GPX activity in HR (*p* = 0.012) and MR (*p* = 0.037) ↓ MDA in MR (*p* = 0.013) and HR (*p* = 0.023) ↔ IL-6, TNF-α and CK.	Azizbeigi et al., 2015 [208]
RE training (6 weeks)	Rats 4 groups: a) RE training b) RE training + alcohol treatment (35% of kilocalorie intake) for 6 weeks c) sedentary d) sedentary + alcohol treatment	6 weeks, 3 days/week Rise onto their hind limbs while wearing lead-weighted vests 30 times per training session	Alcohol treatment in the sedentary animals: ↑ cardiac malondialdehyde, lipid peroxidation ↓ index of myocardial antioxidant potential	Chicco et al., 2006 [209]
RE training (14 weeks)	elderly men and women	14 weeks whole body regular RE	↓ 8-OHdG ↔ Protein content for CuZnSOD, MnSOD, and catalase, and enzyme activities for citrate synthase, mitochondrial ETC complex I+III, and complex II+III	Parise et al., 2005 [210]
CVE training (9 weeks)	Male wistar rats	9 weeks, 5 sessions/week, 60 min/session for 6 weeks and 90 min/session for 3w	↔ TBARS, reactive carbonyl derivatives content, ↓ 8-OHdG ↑ DT-diaphoase and proteasome complex	Radak et al., 1999 [211]

4-HNE: 4-hydroxynonenal conjugated proteins; 8-IsoP: 8-Isoprostane; 8-OHdG: 8-hydroxy-2′-deoxyguanosine; AKT: serine/threonine-specific protein kinase; BMI: body mass index; CAT: catalase; CK: creatine kinase; CSE: cystathionine-γ-lyase expression; CuZnSOD: Copper- and zinc-containing superoxide dismutase; CVE: cardiovascular exercise; eNOS: endothelial nitric oxide synthase; FOXO1: forkhead box protein O1; GPx: glutathione peroxidase; GSH: reduced glutathione; HDL-C: high-density lipoprotein cholesterol; hs-CRP: high-sensitivity C-reactive protein; IL-10: Interleukin-10; IL-6: Interleukin-6; iNOS: inducible nitric oxide synthase; LDH: lactate dehydrogenase; LHP: Lipid peroxidation; LOOHs: lipid hydroperoxides; LOX-1: Lectin-like oxidized low-density lipoprotein receptor-1; MDA: malondialdehyde; MMP: matrix metalloprotease; MnSOD: manganese superoxide dismutase; NO: nitric oxide; NOx: nitrogen oxides; oxPAPC: oxidized 1-palmitoyl-2-arachidonoyl-sn-glycero-3-phosphocholine; RE: resistance exercise; SOD: superoxide dismutase; SOD2: superoxide dismutase 2; *sVCAM*-1: Soluble Vascular Cell Adhesion Molecule-1; TAC: total antioxidant capacity; TBARS: thiobarbituric acid-reactive substances; TNF-α: tumor necrosis factor-α; VO_2_max: maximum oxygen uptake. ↑: significant increase *p* < 0.05 vs. control; ↓: significant decrease vs. pre; ↔: no significant change.
